# Newborn signal functions in Bangladesh: Identification through expert consultation and assessment of readiness among public health facilities

**DOI:** 10.7189/jogh.12.04079

**Published:** 2022-09-17

**Authors:** Shema Mhajabin, Goutom Banik, Muhammad Shariful Islam, Md Jahurul Islam, Tazeen Tahsina, Farid Uddin Ahmed, Mushair Ul Islam, Md Abdul Mannan, Sanjoy Kumer Dey, Samina Sharmin, Fida Mehran, Mahbuba Khan, Anisuddin Ahmed, Ahmed Al Sabir, Shahin Sultana, Ziaul Ahsan, Sayed Rubayet, Joby George, Afsana Karim, Muhammad Shahidullah, Shams El Arifeen, Ahmed Ehsanur Rahman

**Affiliations:** 1 Maternal and Child Health Division, International Centre for Diarrhoeal Disease Research, Dhaka, Bangladesh; 2 Directorate General of Health Services, Government of Bangladesh Ministry of Health and Family Welfare, Bangladesh; 3 Directorate General of Family Planning, Ministry of Health & Family Welfare, Bangladesh; 4 Bangabandhu Sheikh Mujib Medical University, Dhaka, Bangladesh; 5 Unicef, Dhaka, Bangladesh; 6 USAID, Dhaka, Bangladesh; 7 World Health Organization, Dhaka, Bangladesh; 8 RTM International, Dhaka, Bangladesh; 9 National Institute of Population Research and Training, Dhaka, Bangladesh; 10 Ipas, Dhaka, Bangladesh; 11 Save the Children, Dhaka, Bangladesh; 12 National Technical Working Committee, Newborn Health, Bangladesh

## Abstract

**Background:**

This study aimed to identify a set of newborn signal functions (NSFs) that can categorize health facilities and assist policymakers and health managers in appropriately planning and adequately monitoring the progress and performance of health facilities delivering newborn health care in Bangladesh and similar low-income settings.

**Methods:**

A modified Delphi method was used to identify a set of NSFs and a cross-sectional health facility assessment among the randomly selected facilities was conducted to test them in public health facilities in Bangladesh. In the modified Delphi approach, three main steps of listing, prioritizing, and testing were followed to identify the set of NSFs. Then, to finalize the set of NSFs and its variables, a total of five Delphi workshops and three rounds of Delphi surveys were conducted. Finally, 205 public health facilities located in 41 randomly selected districts were assessed for the availability and readiness of finalized NSFs using the updated tool of Bangladesh Health Facility Survey (BHFS) 2017.

**Results:**

Twenty NSFs were identified and finalized, nine of which were categorized as primary NSFs, 13 as basic NSFs, 18 as comprehensive NSFs, and 20 as advanced NSFs. Almost all district hospitals (DHs), Upazila health complexes (UHCs,) and maternal and child welfare centres (MCWCs) performed the primary NSFs in the last three months. However, around one-third of the union health and family welfare centres (UH&FWCs) and very few community clinics (CCs) performed them during the same period. The basic, comprehensive, and advanced NSF readiness was inadequate and inappropriate across all types of facilities, including DHs and UHCs.

**Conclusions:**

In the absence of internationally or nationally agreed-upon NSFs to measure a health facility's service availability and readiness for providing newborn care, this study becomes the first to identify and finalize a set of NSFs and to incorporate relevant variables in the health facility assessment tool which can be used to monitor the availability and readiness of a newborn care facility. The identified NSFs can also be adapted for the countries with similar contexts and can serve as a standard base to determine a global set of NSFs.

In the last decade, commendable progress has been made in improving maternal and newborn health globally. During the Millennium Development Goals (MDG) era (1990-2015), the global neonatal mortality rate (NMR) declined from 36 19 deaths per 1000 live births; the decline in NMR (47%) has been much slower than that of the post-neonatal under-five mortality rate (58%) [1]. With the current trend, the proportional contribution of neonatal deaths among all under-five deaths will increase from 45% in 2015 to 52% in 2030 [2]. Therefore, the Sustainable Development Goals (SDGs) 3.2 set ambitious targets of decreasing newborn deaths to at least 12 deaths per 1000 live births by 2030 [3]. This implies that high-burden countries will have to achieve an average annual rate of reduction (AARR) of 3.7% in the SDG era in contrast to the 2.7% that they have achieved in the MDG era. Bangladesh is one of the South Asian countries with a high NMR of 30 per 1000 live births in 2017 with slower rates of decline. As a result, newborn deaths now account for 67% of all under-five deaths, compared to 57% in 2017 [4].

Considering this high burden, the government of Bangladesh is highly committed to achieving the SDG targets of newborn mortality by 2030. In response to the global Every Newborn Action Plan (ENAP), it developed the Bangladesh Every Newborn Action Plan (BENAP) with specific strategies for averting newborn mortality and morbidity. Following up on these commitments, newborn health has been included as one of the priority activities in the fourth health sector program (2016-2022) of Bangladesh, while the operation plan has a separate programme called the National Newborn Health Programme and Integrated Management of Childhood Illness (NNHP&IMCI) for implementing the newborn health interventions targeting the major causes of newborn death in Bangladesh [5].

Since most of these interventions involve specialized and facility-based treatment, a robust health infrastructure is needed to provide the necessary services [6,7]. Service availability and readiness of health facilities can play a crucial role in the ability of health systems to offer health services to the target population. Inadequate service availability and readiness of health facilities for providing healthy and sick newborn care may act as a significant barrier to achieving effective coverage of these interventions at scale [8,9]. Service availability and readiness of a health facility for delivering specific interventions can be measured through a set of signal functions, which are either the specific skills of the service providers or the availability of specific services through which an intervention can be delivered in a health facility. Unfortunately, to date, there are no globally and nationally recognized newborn signal functions through which the service availability and readiness of a health facility for delivering newborn services can be assessed [10]. Thus, this study identified only eight documents on newborn care signal functions from which it extracted 32 functions and strongly recommended 23 maternal and newborn signal functions.

Emergency obstetric care (EmOC) signal functions (interventions that manage the leading causes of maternal death) have been used to assess the availability and quality of maternal care [11]. The latest incorporation of neonatal resuscitation to these signal functions recognizes the mother-baby treatment continuum [10]. Nonetheless, the provision of neonatal resuscitation alone cannot sufficiently identify a facility's ability to provide routine and emergency newborn care. Moreover, the emphasis on EmOC has been followed by a lack of attention to signal functions for newborn routine health care that may save lives by eliminating complications or intervening before the onset of life-threatening complications [10,12].

A set of signal functions is crucial in planning policy and programs for correctly identifying health facilities that provide newborn services. Acknowledging the necessity and significance, NNHP & IMCI Program of the Directorate General of Health Services (DGHS) has designated the identification of newborn signal functions (NSFs) as a priority task for successful planning and scaling-up of newborn interventions in Bangladesh [13]. This study aimed to identify a set of NSFs, develop a set of indicators and required assessment tools, and assess service availability and readiness of the public health facilities as per NSFs in Bangladesh.

## METHODS

This cross-sectional study was carried out from October 2017 to February 2020. It adopted the Delphi method to seek expert consultation and built consensus for identifying the NSFs and their relevant variables as well as adapting the assessment tool. The Delphi method, mainly developed by Dalkey and Helmer at the Rand Corporation in the 1950s, is well adapted for building consensus due to its evident potential for revealing underlying assumptions and searching out new perspectives that can contribute to consensus among different respondents [14]. The Delphi method uses repeated iterations of ranking surveys and controlled reviews from an expert group to achieve consensus [15]. In contrast to the conventional survey, which focuses on what is, the Delphi survey aims to answer what could or should be [16].

**Figure 1:**
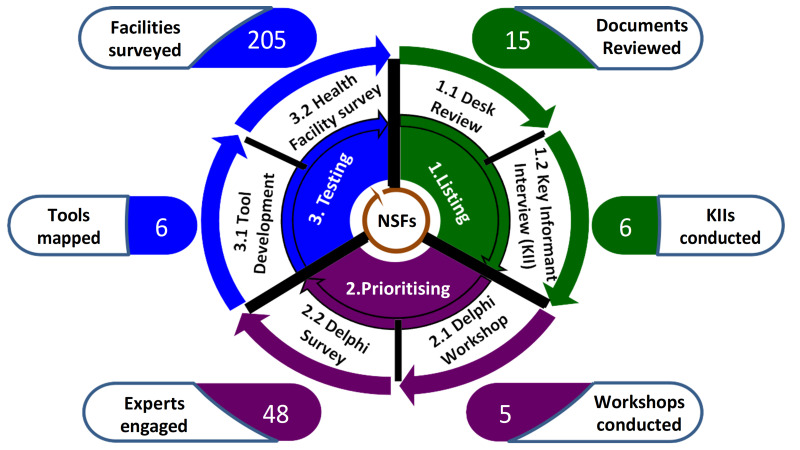
The Delphi steps taken to identify and finalize newborn signal functions (NSFs) of Bangladesh.

Three main steps of the Delphi process (listing, prioritizing, and testing) were followed, with each step being divided into two sub-steps, as illustrated in **Figure 1**.

### Sub-step 1.1: Desk review

To identify the leading causes of neonatal mortality, relevant interventions, indicators, and possible functions (skills and resources needed to provide an intervention), a comprehensive desk review of the available literature (both written and unpublished) was undertaken. We develop a list of existing maternal, newborn, and child health strategies, action plans, monitoring frameworks, standard operating procedures (SOPs), and national and global guidelines on interventions/functions to avert major causes of newborn deaths. A group of International Centre for Diarrhoeal Disease Research, Bangladesh (icddr,b) researchers systematically reviewed relevant five global and ten national documents from the list. Table S1 in the **Online Supplementary Document** includes the documents which were reviewed to develop a list of 79 newborn interventions/functions.

### Sub-step 1.2: Key informant interviews

Besides desk review, a total of six key informant interviews (KIIs) were conducted with Ministry of Health & Family Welfare (MoHFW) program managers and newborn health experts from professional bodies such as Bangabandhu Sheikh Mujib Medical University (BSMMU) and Bangladesh Neonatal Forum (BNF) to identify additional interventions and functions needed to deliver newborn care in a health facility setting. The interviews helped to identify an additional 11 newborn interventions/functions that were added to the primary list of 79 newborn interventions/functions.

### Sub-step 2.1: Delphi workshops

The National Technical Working Committee for Newborn Health (NTWC-NBH) (established by MoHFW) formed a group of newborn expert panels with a specific Term of Reference (ToR). A total of 48 newborn experts, representing 22 organizations such as the MoHFW, professional bodies, United Nation (UN) agencies, and development partners engaged in newborn health, became part of the newborn expert panel (Figure S1 in the **Online Supplementary Document**). The newborn expert panel members were invited to participate in five Delphi workshops and three rounds of Delphi surveys held from October 2017 to June 2019 to finalize the NSFs and their variables for Bangladesh (Figure S2 in the **Online Supplementary Document**). During the workshops, the newborn expert panel members prioritized 37 of the proposed 90 newborn interventions/functions. The 37 prioritized newborn interventions/functions were scored against the four NSFs categories (primary, basic, comprehensive, and advanced) by the newborn expert panel members during the Delphi surveys. The scoring was done on a four-point Likert scale (1 = not important, 2 = moderately not essential, 3 = moderately essential, and 4 = essential).

### Sub-step 3.1: Development of the health facility assessment tool for the final set of newborn signal functions

#### Variable mapping

The variables required for each NSFs were identified through an extensive desk review of existing six health facility assessment (HFA) tools: Averting Maternal Death and Disability (AMDD), Health Facility Census (HFC), Service Provision Assessment (SPA), Rapid Health Facility Assessment (R-HFA), Service Availability Mapping (SAM), and Service Availability and Readiness Assessment (SARA) [10]. The 2017 Bangladesh Health Facility Survey (BHFS) tool was the base document for this mapping exercise. The 2017 BHFS tool provided 45 relevant variables and the other HFA tools an additional 16 variables. Consultation with the newborn Delphi experts helped us to identify 20 more variables. Therefore, a total of 81 essential variables were listed and mapped for the final set of NSFs.

#### Validation of the identified variables

The variables present in the 2017 BHFS tool (n = 45) and the variables present in the other HFA tools (n = 16) were automatically included in the final variable list. Variables identified from interviews with the newborn Delphi experts were validated before they were included in the final variable list. Project research physicians (PRPs) measured the variables’ construct and content validity by field testing them in seven different public health facilities. The operational definition of these validity measures is described in the published protocol [13]. Content validity was analysed using descriptive statistics and it was considered achieved when more than 80% of the newborn Delphi experts agreed that the language of the newly added question appropriately reflects the content/variable that it was supposed to measure. Kappa statistics were used to report on the construct validity of the newly added variables (data collector vs PRPs). Construct validity of the variable was achieved if the kappa value was ≥0.75.

#### Tool finalization

The questionnaire adaptation for the 2017 BHFS tools took place in August 2019. A consultative meeting with the stakeholders especially from National Institute of Population Research and Training (NIPORT), visits to the service provision sites for pre-testing the tools, and a day-long questionnaire adaptation workshop elicited the feedback needed to adapt the questionnaires. Based on the feedback, the validated variables were incorporated into the BHFS 2017 tool, and finally, the tool was updated and finalized for the assessment of service availability and readiness of NSFs in Bangladesh. The updated 2017 BHFS had two types of data collection tools as facility Inventory questionnaire and the health care provider interview questionnaire.

### Sub-step 3.2: Conducting health facility assessment

#### Training of the data collectors

12 PRPs were recruited for the HFA who received five days of training on the updated BHFS 2017 tool in September 2019, in Dhaka.

#### Data collection and data quality monitoring

Four data collection teams were formed, with one data collector on each team assigned the role of a team leader. Data collection was conducted from November to December 2019. On average, data collection took two days for each health facility. And was conducted using paper-based Facility Inventory and Health Care Provider Interview questionnaires. The data collectors interviewed the most knowledgeable person about the facility and its services; if collected from other persons, the information was validated by direct physical observation and/or some facility record review.

Two medical doctors monitored the overall data collection process and supervised the team. They conducted periodic visits to ensure the quality of data collection and provide onsite training.

**Figure 2:**
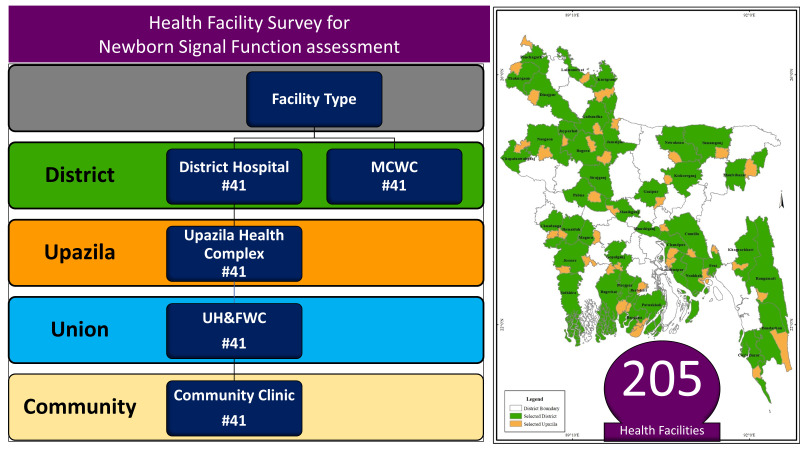
The number and type of public health facilities that were assessed to test the functionality of identified newborn signal functions (NSFs) in Bangladesh.

To assess the service availability and readiness of the public health facilities for the NSFs, a precision-based sample size was measured. With a 10% error margin, the study aimed to cover maximum variance (50%) as there is no estimate available. The required sample size was 101 public health care facilities, assuming a non-response rate of about 5%. We were able to conduct a cross-sectional facility assessment in 205 registered public health facilities across all eight divisions to provide the most representative results for Bangladesh (**Figure 2**).

#### Variable mapping

The variables required for each NSFs were identified through an extensive desk review of existing six health facility assessment (HFA) tools (AMDD, HFC, SPA; R-HFA, SAM, and SARA [10]).

### Data management and data analysis

To analyse each Delphi survey outcome, central tendency (mean) and a measure of distribution (interquartile range (IQR)) were measured. Additionally, the average expert agreement (AEA) for every intervention/function was calculated from the ratio between the frequency of mode and the number of responses in each survey. As experts recommended, consensus and agreement were stable when the AEA value was 50 or more for any intervention/function. Kappa statistics with a >0.75 cut-off were used to report on the construct validity of the newly added variables in the HFA tool. The BHFS tabulation plan was followed to analyse the HFA data.

### Ethical considerations

Ethical approval was obtained from the Research Review and Ethical Review Committee (ERC) of icddr, b (PR-17089). Written and informed consent was sought from the health facilities and each participant before the assessment or interviews.

## RESULTS

**Figure 3:**
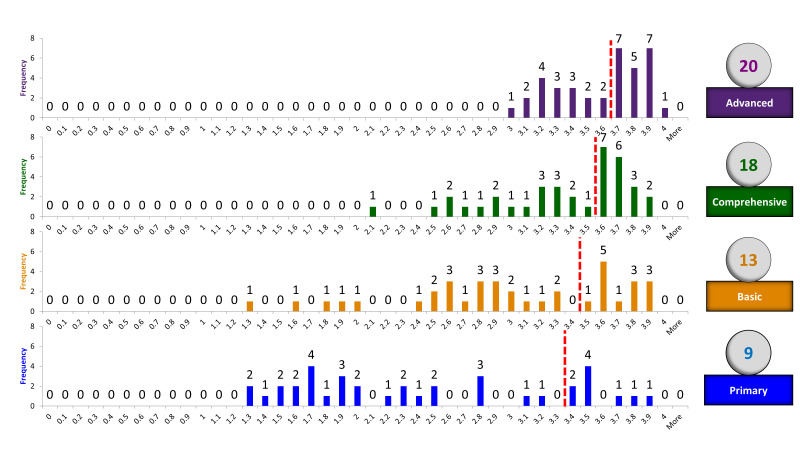
Mean Delphi score of experts for the prioritized Newborn Signal Functions (NSFs) as per the cut-off value.

Based on the pre-defined cut-off values and scoring guidelines recommended by the newborn Delphi experts, we identified 20 NSFs from the shortened list of 37 interventions/functions for Bangladesh. The summary of the average score for each intervention/function and the cut-off values to identify four categories of NSFs is presented in **Figure 3**. Nine interventions/functions were identified as primary NSFs when the cut-off value was an average score of 3.3 (**Figure 3**). Then, fixing an average cut-off value of 3.4 for basic NSFs, a total of 13 interventions, including nine primary NSFs, were found. Furthermore, an average cut-off value of 3.5 provided 18 comprehensive NSFs which included 13 basic NSFs. Finally, when the average cut-off value was set at 3.6, a total of 20 advanced NSFs were identified, including all primary, basic, and comprehensive NSFs (Figure S3 in the **Online Supplementary Document**).

**Figure 4:**
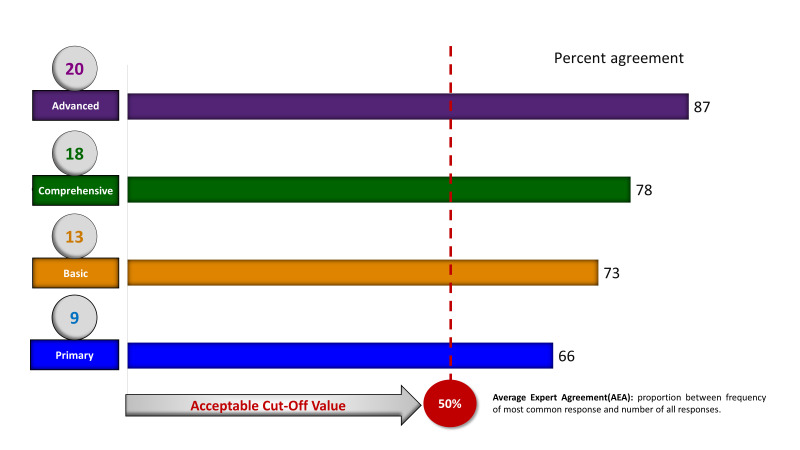
The average expert agreement (AEA) of the Delphi experts for each category of identified newborn signal functions (NSFs) as per the cut-off value.

The AEA of the Delphi experts on newborns was checked based on each category of NSFs on the pre-defined cut-off values (**Figure 4**). It showed that the AEA among the experts was more than 50% across all categories of NSFs. The highest percentage of AEA was found for advanced NSFs (87%), and the lowest percentage of AEA was found for primary NSFs (66%). Therefore, most of the newborn Delphi experts reached the pre-defined threshold of consensus.

The HFA data provided the status of identified NSFs functionality in the selected public health facilities of Bangladesh which is presented in **Table 1**. The key findings for each NSF have been explained below:

**Table 1:** Percent of facilities performed identified newborn signal functions in the last three months (functionality) by type of facilities

Newborn signal functions	DH (%)	MCWC (%)	UHC (%)	UH&FWC (%)	CC (%)	Total (%)
**Primary**						
Iron and folic acid supplement	93	100	100	59	10	72
Hand washing before touching baby	100	100	100	54	10	73
Immediate drying	100	100	100	54	10	73
Delayed cord clamping	100	100	100	54	10	73
Clean cord-cutting	98	98	98	54	10	71
Application of 7.1% CHX for cord care	100	100	100	54	10	73
Resuscitation – bag and mask	98	100	93	46	5	68
Early initiation of breastfeeding	98	100	100	51	10	72
Early initiation of skin-to-skin care	100	100	100	54	10	73
All primary NSFs (n = 9)	85	98	90	39	5	63
**Basic** (Includes 9 NSFs above))						
Tetanus toxoid	85	95	81	27	2	58
Suction of newborn	100	95	98	44	2	68
Oral antibiotics for infections	23	15	20	5	0	12
Injection antibiotics (IM) for infections	48	34	37	0	0	24
All basic NSFs (n = 13)	13	7	12	0	0	6
**Comprehensive (includes 13 NSFs above)**						
Oral or injection antibiotics for PROM	98	73	76	0	0	50
Oxygen therapy	98	100	95	0	0	60
Injection antibiotics (IV) for infections	78	39	56	0	0	35
Phototherapy	88	2	10	0	0	20
KMC	63	39	29	0	0	28
All comprehensive NSFs (n = 18)	10	0	0	0	0	2
**Advanced (includes all NSFs above)**						
Injection ACS for preterm birth	78	56	51	0	0	37
Incubator	50	0	2	0	0	10
All advanced NSFs (n = 20)	5	0	0	0	0	1

NSF – newborn signal function, DH – district hospital, MCWC – maternal and child welfare centres, UHC – Upazila health complex, UH&FWC – union health and family welfare centre, CC – community clinic, IV – intravenous, IM – intramuscular, KMC – kangaroo mother care, CHX – chlorhexidine

### Iron and folic acid supplementation

Less than two-thirds of the facilities performed iron and folic acid (IFA) supplementation function. Almost all district hospitals (DHs), Upazila health complexes (UHCs,) and maternal and child welfare centres (MCWCs) provided IFA supplementation in the last three months, while around one-third of union health and family welfare centres (UH&FWCs) and less than 10% of community clinics (CCs) provided IFA supplementation in the same period.

### Hand washing each time before touching the baby

One-fourth of the facilities did not perform hand washing each time before touching the baby. All DHs, MCWCs, and UHCs performed hand washing each time before touching the baby, whereas around half of the UH&FWCs and less than 10% of CCs performed hand washing each time before touching the baby.

### Immediate drying

About 73% of the facilities performed immediate drying. Moreover, all DHs, MCWCs, and UHCs performed the immediate drying of the newborn, while around half of the UH&FWCs and less than 10% of CCs performed this function.

### Delayed umbilical cord clamping approximately one to three minutes after birth

About 73% of the facilities performed delayed umbilical cord clamping approximately one to three minutes after birth. All DHs, MCWCs, and UHCs performed delayed umbilical cord clamping approximately one to three minutes after birth, whereas the function was performed by around half of the UH&FWCs and less than 10% of CCs.

### Clean cord-cutting (clean/sterile thread)

About 72% of the facilities performed a clean cord-cutting function. Nearly all DHs, MCWCs, and UHCs performed clean cord-cutting. However, around half of the UH&FWCs and less than 10% of the CCs performed this function.

### A single application of 7.1% chlorhexidine to the umbilical cord followed by dry cord care

About 73% of the facilities performed a single application of 7.1% chlorhexidine to the umbilical cord followed by dry cord care. All DHs, MCWCs, and UHCs performed the single application of 7.1% chlorhexidine to the umbilical cord followed by dry cord care, while only around half of the UH&FWC and less than 10% of the CCs performed this function.

### Neonatal resuscitation

About 68% of the facilities performed neonatal resuscitation. All MCWCs, nearly all DHs, and about 93% of the UHCs performed this function.

### Early initiation of breastfeeding within one hour

About 72% of the facilities performed early initiation of breastfeeding within one hour. All MCWCs and UHCs performed this function whereas 2% of DHs did not perform this function.

### Skin-to-skin care for two hours

About 73% of the facilities performed skin-to-skin care for two hours. While all DHs, MCWCs, and UHCs performed the clean cord-cutting (clean/sterile thread) function, only 53% of the UH&FWC and 10% of the CCs performed this function.

### Tetanus toxoid

Only 58% of all facilities performed tetanus toxoid function. The function was performed by over 80% of the DHs, MCWCs, and UHCs, but by less than one-third of the UH&FWCs.

### The suction of newborns

One-third of the total facilities performed suction of newborns. All DHs and nearly all UHCs and MCWCs performed this function, but less than 50% of the UH&FWCs performed it.

### Oral antibiotics for infections

Only 12% of the total facilities provided oral antibiotics for infections. Across facilities, the performance of this function was lower than 25%, and no CCs performed this function.

### Injectable antibiotics (intramuscular) for infections

Only 24% of the total facilities provided injectable antibiotics (intramuscular (IM)) for infections. Besides, none of the UH&FWCs and CCs performed this function.

### Antibiotics for premature rupture of the membrane

Only half of the total facilities provided antibiotics for premature rupture of the membrane (PROM). Nearly all the DHs provided antibiotics for PROM, while none of the UH&FWCs and CCs performed this function.

### Oxygen therapy

About 60% of the total facilities provided oxygen therapy. However, only 78% of the DHs, 56% of the UHCs, and 39% of the MCWCs performed this function.

### Injectable antibiotics (intravenous) for infections

Only 35% of the total facilities provided injectable antibiotics (intravenous (IV)) to the newborn. Besides only 78% of the DHs, 56% of the UHCs, and 39% of the MCWCs performed this function.

### Phototherapy

Less than 30% of the total facilities provided phototherapy. Moreover, about 88% of the DHs and less than 10% of the UHCs and MCWCs performed this function.

### Kangaroo mother care for preterm and low birth weight babies

Only 28% of the total facilities provided kangaroo mother care (KMC) for pre-term and low birth weight (LBW) babies. Only 63% of the DHs, 39% of MCWCs, and less than one-third of the UHCs performed this function.

### Antenatal Corticosteroids (ACS) for preterm birth

Only 37% of the total facilities provided ACS for preterm birth. Only 78% of the DHs and around 50% of the MCWCs and UHCs performed this function.

### Incubator

Only 10% of the total facilities performed incubator functions. Only half of the DHs and 2% of the UHCs performed this function, while none of the MCWCs performed this function.

Overall, almost all the MCWCs, 90% of the UHCs, and 85% of the DHs performed all primary NSFs, while only 39% of the UH&FWCs and 5% of the CCs performed those in the previous three months of the assessment. Only 5% of DHs performed advanced NSFs.

## DISCUSSION

Using the adapted Delphi method, a clear, evidence-based, and low-cost technique combining scientific methods and expert opinion [17-19], this study identified 20 NSFs in four categories (primary, basic, comprehensive, and advanced) and their variables by reaching a pre-defined consensus level among the newborn Delphi experts. The NSFs variables were then incorporated into the BHFS 2017 tool to measure the service availability and readiness of the public health facilities after testing their validity. The adapted BHFS 2017 tool was successfully implemented in 205 public health facilities in Bangladesh which provided data regarding the status of NSFs in those facilities. This study found poor functionality of each primary NSFs at UH&FWCs and CCs whereas it was nearly present in all DHs, MCWCs, and UHCs. The functionality of all primary, basic, comprehensive, and advanced NSFs was higher in DHs compared to other facilities.

In identifying a set of valid and agreed-upon NSFs, this study followed all relevant steps of a Delphi study including defining consensus. A Delphi study must have a pre-defined meaning of consensus for their study, if not, the credibility of the outcomes will not be considered as valid [20]. Various Delphi studies have measured consensus in different ways: by a predetermined number of rounds, subjective analysis, a certain level of agreement, the average per cent of majority opinions cut-off rate, mode, mean, median ratings and rankings, interquartile range, coefficient of variation, and post-group consensus [18,20-24]. As there is no agreed-upon approach to measure the consensus level, some of the measures are commonly used and adapted based on context and requirement. Additionally, there is no accepted, set standard for the target percentage of agreement, and 70% (summative of agreeing and strongly agree) is commonly reported in the literature [24,25]. Therefore, using more than one measurement of consensus helps with reaching better conclusions. In this study, a four-point Likert scale was used for participants to rate their level of agreement or disagreement for each intervention/function. To measure the consensus, this study utilized the mean score and the AEA. The minimum cut-off for the mean score was 3.3, while consensus and agreement among the newborn Delphi experts were considered stable if the AEA value was 50. Since we wanted the expert panel to either select or reject an intervention/function as a signal function, we used an even number Likert Scale to avoid neutral positions. Each member of the expert panel was asked to assign a score for each of the interventions/functions. Five- or seven-point Likert Scale are usually used in Delphi studies to allow the participants to take a neutral position [26].

The utilization of RAND-modified Delphi in this study improved the validity and generalisability of the identified NSFs [27]. The response rates for each survey were high, with 100% in the first Delphi survey and 85% in the other two surveys. A total of 48 Delphi experts representing 22 organizations (government, academia, professionals, service providers, and development partners) participated in this study. This heterogeneity brought a wealth of experience and knowledge and enhanced the discussions. However, unlike some other Delphi studies, this study did not have an international expert panel because it aimed to finalize the NSFs for Bangladesh [10,22]. The Delphi experts involved in this study had knowledge and experience of global newborn health status. This study also engaged the newborn Delphi experts during the workshops and surveys for over two years, which increased the cost and took time. Organizing workshops and/or preparing for the surveys took several weeks of preparation which required strong commitment and patience. In addition, intensive resources including the person engaged in the planning, data collection, data analysis, and other activities were utilized. Anonymity was maintained throughout the scoring process, protecting the individual’s opinion when prioritizing the NSFs by keeping aside any stakeholders’ power dynamics that could have suppressed individual opinions. The novel and rigorous approach ensured that the newborn Delphi experts’ engagement and consensus identified the NSFs based on the content, context, and scalability. Hence, the identified NSFs can provide a strong base for similar low- and middle-income countries to start with and they may require a lower number of expert engagement activities such as workshops and surveys.

The identified valid NSFs can capture the service availability, readiness, and functionality of a range of relevant newborn interventions/functions at different facility levels, which can help identify the causes of the high burden in newborn mortality and the recent halt in the reduction in newborn morbidity. In this study, a total of 20 interventions were accepted and 17 were rejected, with a focus on impact, reliability, actionability, priority, and feasibility of data collection during the scoring and ranking process. A similar attempt undertaken in Zimbabwe identified 11 obstetric and newborn signal functions, three of which were for routine newborn care, six for basic emergency newborn care, and two functions for comprehensive emergency newborn care [10]. To finalize the obstetric and newborn signal functions, the study conducted an online survey where only 39 international experts from low-income countries participated. Out of 32 interventions, a total of 23 interventions received a high percentage, but only 11 were presented as final newborn functions. The prioritization and finalization process lacked lucid details and criteria. On contrary, this study rigorously utilized the novel Delphi method and conducted multiple rounds of workshops and surveys to achieve the consensus of 48 national newborn experts from various backgrounds and organizations, including the government. Instead of a one-time survey, we continuously engaged the experts, beginning from sensitization to consultation, for nearly two years. However, our study includes all the signal functions that the Zimbabwe study presented except alternative feeding if the baby is unable to breastfeed. It refers that our study has generated a comprehensive list of NSFs which can be adapted to similar country contexts and globally.

These 20 identified NSFs can be captured as part of a health facility assessment survey or as a routine monitoring system by health managers at any health facility level. When reviewing existing HFA tools, we found that most NSFs are not collected through health facility surveys. The 2017 BHFS tool only measured the availability and readiness of one newborn indicator (newborn resuscitation) as part of Basic Emergency Obstetric Care (BEmOC) of public and private health facilities in Bangladesh, which was insufficient to accurately measure the health facility's availability and readiness to provide newborn care and to assess the status of identified NSFs. All these NSFs are not routinely collected or reported in the existing health information system. Having an agreed list of functions made it relatively easy to identify and finalize the list of variables required to measure the service availability and readiness of these functions at a health facility level. The results of the survey conducted by the updated HFA tool contributed to assessing the health facilities for the NSFs and will assist policymakers and health managers in planning and monitoring their performance and progress regarding newborn health services [28]. The previous study did not develop or adapt any HFA tool to assess the NSFs [10].

The HFA of this study revealed that the functionality of NSFs for neonatal infection management such as oral antibiotics, injectable antibiotics (IM), and injectable antibiotics (IV) was poor, especially in primary referral facilities. Infections such as sepsis, pneumonia, tetanus, and meningitis account for about 22% of overall newborn deaths worldwide and around one-quarter of newborn deaths in Bangladesh [29,30]. The World Health Organization (WHO) recommends inpatient hospital care for all neonates with infection treatment with multi-drug multi-dose oral and injectable antibiotics [31]. Three-quarters of neonatal deaths occur in the first week of life and about one million newborns die within the first 24 hours [32]. Immediate newborn care is crucial for the survival of neonates. Even though immediate essential newborn care such as immediate drying, delayed cord clamping, clean cord-cutting, skin-to-skin care, early initiation of breastfeeding, neonatal resuscitation, and suction of newborn is considered important in reducing a newborn’s adverse health outcomes [33], the availability of these interventions was also inadequate, especially in primary level facilities. Improving care for small and ill newborns has been overlooked in the past, although it could avert an estimated 600 000 infant deaths per year around the world [34]. However, our survey revealed inadequate availability of oxygen therapy, phototherapy, KMC, ACS, and incubation at almost all health facility levels, thus, contributing to 46% of neonatal death within 24 hours and 86% of neonatal death in the first week of life in Bangladesh [35]. The overall findings of a cross-sectional HFA helped us identify existing gaps in ranges of newborn interventions/functions and can guide us in more effectively and timely averting newborn mortality and morbidity if we can employ routine assessment of the health facilities as per the NSFs.

This research study and its findings primarily aimed to influence national policy and programs. From the study’s initiation, the NNHP & IMCI Program of the DGHS was co-leading the study activities with icddr,b [36]. All the Delphi workshops were conducted at the conference centre of NNHP&IMCI; DGHS and all members of NNHP&IMCI actively participated in the workshops and surveys. The government stakeholders’ interest also raised the interest and commitment level of other stakeholders. The identified NSFs were endorsed by NNHP&IMCI, MOH&FW, professional bodies (Bangladesh Paediatric Association, Bangladesh Neonatal Forum, Bangladesh Perinatal Society, Obstetrical and Gynaecological Society of Bangladesh), and the 48 national technical experts who participated in the study. The government incorporated the NSFs into the NNHP monitoring checklist to monitor newborn health care practices in union facilities and Upazila and above-level health facilities [37]. NNHP & IMCI sent a letter to the union and Upazila and above-level facilities to report newborn health service practices of monitoring checklist routinely. Moreover, NNHP & IMCI developed an electronic version of the monitoring checklist and provided training to around 500 health managers across the country. The e-version of the monitoring checklist is being filled up from union to above-level facilities in each quarter. Based on the findings from the monitoring checklist, NNHP & IMCI is organizing divisional progress review workshops in participation with divisional level managers to discuss the health facilities’ performance of that division and is taking necessary initiatives to overcome the shortfalls. The government also planned to integrate the monitoring checking as a dashboard in DHIS2 for real-time performance monitoring and instant decision making, while recommending the use of NSF as a planning and monitoring tool in the National Newborn Health Strategy and National Child Health Strategy. The NSFs have been used as a planning tool in the mid-term review of the 4th health sector program, and a decision was made to further use NSF as a planning tool in the 5th health sector program. The acceptance and endorsement of the NSFs at the national level were possible due to the technical expertise and experience of icddr,b in Bangladesh, and pre-existing relationships with other stakeholder organizations. Moreover, the communication and organizing skills of icddr,b contributed to facilitating the stakeholder engagement process efficiently.

Ten years have left to achieve the ambitious Sustainable Development Goals (SDGs) of ending preventable neonatal deaths, accelerated efforts are needed to improve access to and quality of care for all mothers and newborns [1-3]. As most newborn interventions are facility-based, these interventions need to be monitored routinely in the health facilities [6,7]. All young infants require basic primary health care and follow–up, including immunization, exclusive breastfeeding assistance, assessment of growth, and developmental surveillance. However, countries yet do not have the standard list of newborn indicators and lack facility assessment tools to measure and monitor services delivered to newborns. The NSFs identified using the widely accepted Delphi method can be used in countries with similar contexts or can be adapted for other countries. Moreover, when endorsed and accepted by the government, the status of newborn interventions can be routinely captured, which can help improve the status of newborns and may help countries achieve the ambitious SDG targets in the remaining time because the household surveys cannot measure the quality with which the interventions are delivered.

### Strengths and limitations

This is the first study to identify widely agreed upon NSFs for Bangladesh by adopting the validated and widely used Delphi methodology to synthesize experts’ knowledge and experiences. The MOHFW of Bangladesh, various associations, academics, and development partners have endorsed the identified NSFs. The existing health facility assessment tool has been updated to test the NSFs in public health facilities for further use. In the future, the updated health facility assessment tool will be used for the national health facility assessment in Bangladesh.

This is a resource-intensive and long-time study. It took us longer than anticipated to ensure the participation of numerous Delphi experts during each workshop and survey. Some Delphi experts were unable to participate throughout the whole process.

## CONCLUSIONS

There are currently no internationally or nationally recognized signal functions that are widely accepted and can be used to measure a health facility's service availability and readiness to provide newborn care. We developed a widely agreed-upon set of NSFs that assisted in updating the national HFA tool. The findings of the survey helped to determine whether health facilities are adequately ready to provide newborn health care services. The results contributed to categorizing newborn care health facilities as primary, basic, comprehensive, and advanced. The identification of the NSFs will guide health managers and policymakers towards appropriate planning and adequately monitoring of the progress and performance of health facilities delivering newborn health care in Bangladesh.

## Additional material


Online Supplementary Document
