# Supplementary material for: Newborn signal functions in Bangladesh: Identification through expert consultation and assessment of readiness among public health facilities
Source: J Glob Health. 2022 Sep 17;12:04079. doi: 10.7189/jogh.12.04079 (PMC9480864; doi:10.7189/jogh.12.04079)

Table S1: List of documents studied during the desk review to identify newborn interventions/functions

Type	Document titles	Document agency
Global	Every Newborn - An Action Plan to End Preventable Deaths, June 2014	WHO; UNICEF
Global	CDC Global Maternal and Child Health Strategy, 2013-2016	US Department of Health and Human Services; Centers for Disease Control and Prevention
Global	Global Strategy of Women's and Children's Health, September 2010	United Nations
Global	The Integrated Global Action Plan for the Prevention and Control of Pneumonia and Diarrhoea (GAPPD), WHO and UNICEF, 2013	UNICEF; WHO
Global	Monitoring Emergency Obstetric Care: a handbook by WHO, UNFPA, UNICEF and AMDD	WHO; UNFPA; UNICEF; Averting Maternal Death and Disability (AMDD)
National	Strategic Investment Plan for Health Population & Nutrition Sector Development Program (HPNSDP), 2017-2022	Planning Wing, Ministry of Health and Family Welfare (MoHFW), Government of the People's Republic of Bangladesh
National	Program Implementation Plan for Health Population & Nutrition Sector Development Program (HPNSDP), 2017- 2022	Planning Wing, MoHFW, Government of the People's Republic of Bangladesh
National	Operations Plan for Health Population & Nutrition Sector Development Program (HPNSDP), 2017-2022	MoHFW, Government of the People's Republic of Bangladesh
National	Essential Service Package for 2017-2022	MoHFW, Government of the People's Republic of Bangladesh
National	National Neonatal Strategy Bangladesh 2009	MoHFW, Government of the People's Republic of Bangladesh
National	Committing to Child Survival: A Promise Renewed Declaration, 2014	UNICEF
National	Bangladesh Every Newborn Action Plan, 2015	MoHFW, Government of the People's Republic of Bangladesh; DGHS; USAID; Save the Children; icddr,b; UNICEF
National	Maternal Health Strategy Bangladesh 2017	DGHS; DGFP; MoHFW, Government of the People's Republic of Bangladesh
National	National Immunisation Policy 2013	DGHS; MoHFW, Government of the People's Republic of Bangladesh

National	Bangladesh National Nutrition Service 2015	MoHFW, Government of the People's Republic of Bangladesh

Figure S1: The organizations endorsed the identified NSFs of Bangladesh

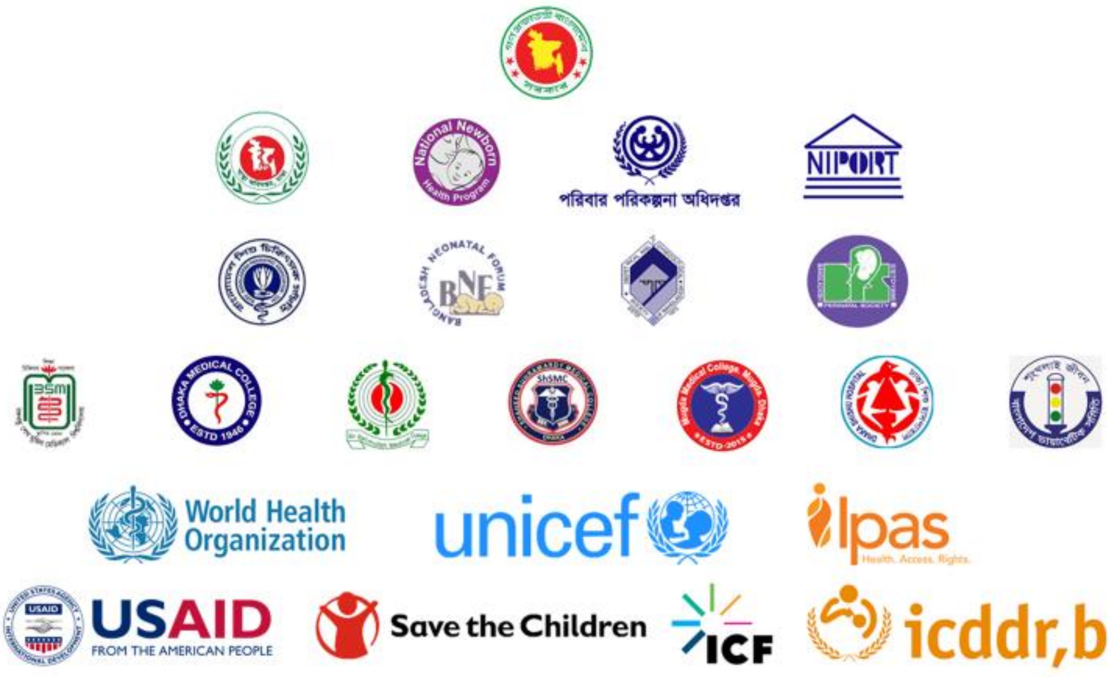

Figure S2: The key activities with time for finalizing the NSFs of Bangladesh

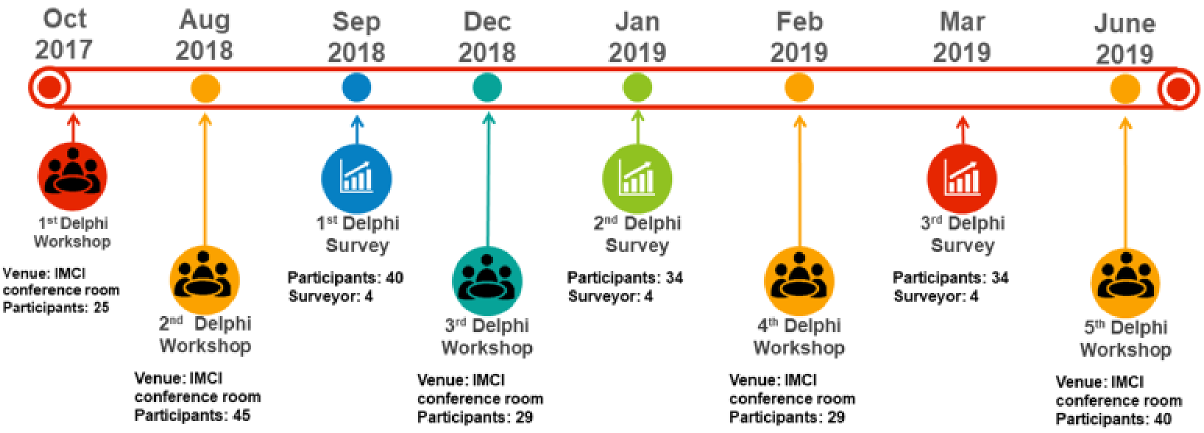

Figure S3: The final set of NSFs of Bangladesh

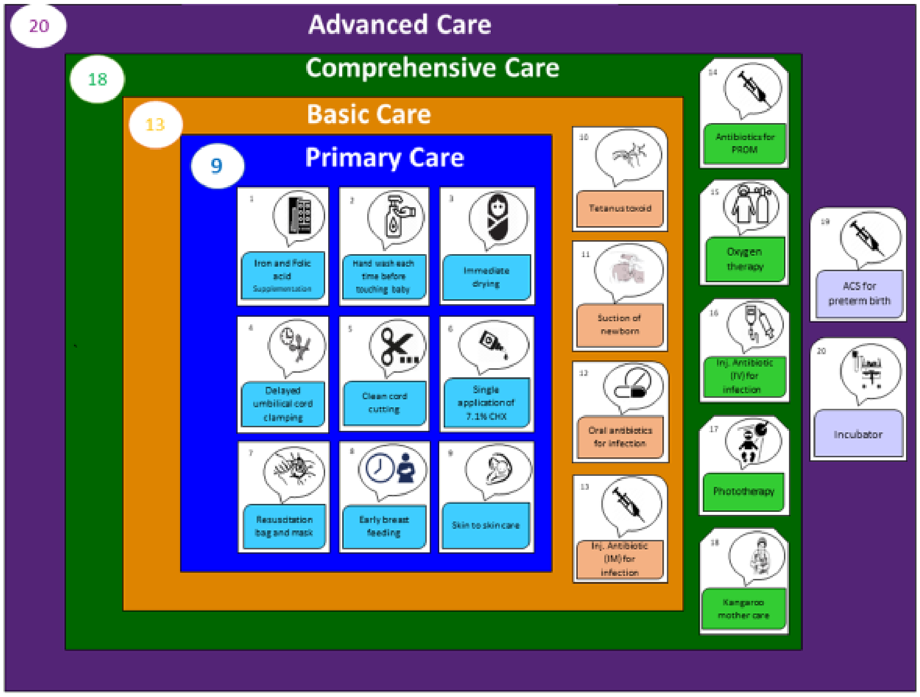

Supplement: Online Supplementary Document [file jogh-12-04079-s001.pdf]
